# The distinct contribution of sternotomy to the systemic inflammatory response during children's heart surgery

**DOI:** 10.3389/fimmu.2025.1617524

**Published:** 2026-01-02

**Authors:** Joel David Bierer, Julia Paffile, Roger Stanzel, Mark Henderson, John Sapp, Pantelis Andreou, Jean Sylvia Marshall, David Horne

**Affiliations:** 1 Division of Cardiac Surgery, Dalhousie University, Halifax, NS, Canada; 2 Undergraduate Medical Education, Faculty of Medicine, Dalhousie University, Halifax, NS, Canada; 3 Department of Clinical Perfusion, Nova Scotia Health Authority, Halifax, NS, Canada; 4 Division of Cardiology, Dalhousie University, Halifax, NS, Canada; 5 Department of Community Health and Epidemiology, Dalhousie University, Halifax, NS, Canada; 6 Department of Microbiology and Immunology, Dalhousie University, Halifax, NS, Canada

**Keywords:** sternotomy, incision, cardiopulmonary bypass, pediatric cardiac surgery, congenital heart disease, complement, cytokine, chemokine

## Abstract

**Background:**

Sternotomy provides access to the mediastinum, heart and great vessels for congenital cardiac surgery in children. The contribution of this incision to the systemic inflammatory response during open-heart surgery, particularly in combination with the complement-mediated response to cardiopulmonary bypass (CPB), is unknown. This study aimed to characterize the inflammatory mediator profile of sternotomy and contrast that with CPB-associated inflammation.

**Methods:**

This study is a *post-hoc* analysis of a single-arm prospective clinical study (NCT05154864) of 40 pediatric patients undergoing congenital cardiac surgery with CPB. Arterial blood samples were taken before and after sternotomy, but before CPB initiation (sternotomy phase), and after CPB exposure (CPB phase). Thirty-three inflammatory mediators from the cytokine, chemokine, complement, and adhesion molecule families were measured. The mediator changes were calculated for each phase and described using median fold changes. A principal component analysis with hierarchical clustering (PCA-HCPC) was conducted on mediator changes over the sternotomy phase.

**Results:**

Compared to baseline, all 16 cytokines and chemokines assessed increased through the sternotomy phase, while complement and adhesion molecules were static or decreased. The most active mediators were IL-1β (3.3x median fold increase), CXCL2 (3.3x), IL-6 (2.6x), IL-10 (2.6x), GM-CSF (2.3x), IL-1α (2.2x) and IL-2 (1.7x). The PCA-HCPC showed three statistically significant clusters, cluster 1 grouped cytokines and chemokines with the sternotomy phase, while complement mediators and adhesion molecules were in separate clusters. In contrast to the CPB exposure, sternotomy showed a predominant contribution of TNF, IL-1α, IL-1β, IL-2, TRAIL, CCL3, CCL4, CXCL1, CXCL2 and GM-CSF to the systemic inflammatory response.

**Conclusions:**

Sternotomy and related tissue trauma produce a distinct systemic inflammatory mediator profile, consisting of pro-inflammatory cytokines and chemokines but not complement. The mediators IL-6, CXCL8, IL-1Ra and IL-10 are sequentially induced by both sternotomy and CPB, representing sequential immunologic stimulation during the cardiac operation.

## Introduction

Cardiac surgery is associated with systemic inflammatory response syndrome (SIRS) that can feature cardiovascular, pulmonary, neurologic, splanchnic, hematologic, and immune system dysfunction, which directly influences the post-operative recovery (1, 2). The inflammatory response arises from many stimuli during the surgery, including tissue trauma, exposure to non-endothelialized cardiopulmonary bypass (CPB) circuit, ischemia-reperfusion, and hypothermia, yielding circulating pro-inflammatory mediators (2). This SIRS from cardiac surgery remains a clinical challenge as effective immunomodulatory therapies remain elusive, probably because of the diversity of immunologic pathways induced through the various stimuli (3, 4). A systematic understanding of the different immunologic reactions is paramount for future innovation and the development of efficacious treatments.

Two prominent immunologic stimuli during open-heart surgery include sternotomy and CPB. Sternotomy is performed at the beginning of open-heart surgery to access the patient's mediastinal and cardiac structures through skin and subcutaneous tissue incision, sternal division and pericardial entry (5). For neonates and young infants, a partial or complete thymectomy is also routinely performed to facilitate adequate visualization of the mediastinal structures (6). Acute immunologic reaction to tissue trauma includes hemostasis, which provides scaffolding for leukocytes, and inflammation, which are most relevant to the peri-operative setting (7, 8). Pro-inflammatory cytokines and chemokines – TNF, IL-1, IL-6, CCL2, CCL3, CXCL1, CXCL2, CXCL8, CXCL8, GM-CSF – are all upregulated in the acute inflammatory phase to stimulate leukocyte recruitment to the incision (7, 8).

CPB, initiated after sternotomy, is essential for open-heart surgery as the extracorporeal circuit provides circulation and oxygen delivery for the patient while providing a bloodless field for the surgeon (9). CPB activates the alternative complement pathway through the C3 roll-over mechanism, cleaving C3 into C3a and C3b, and subsequently releasing various cytokines and chemokines (10). C3b is a major player in the initiation, amplification, and effector functions of the whole complement pathway (10). Once CPB initiates the inflammatory cascade, this leads to an acute phase with pro-inflammatory reactions but also triggers a later resolution phase with anti-inflammatory interleukins 10 (IL-10) and receptor antagonist IL-1Ra (10). Importantly, the complement mediators (specifically C2, C3, C3a, C3b, C5, and C5a) have been shown to correlate with adverse outcomes after cardiac surgery, whereas the relevance of IL-6 and CXCL8 remains controversial (2, 11).

There has been limited research on the contribution of sternotomy to overall inflammatory sequelae in open cardiac surgery requiring CPB. Only a few human studies have investigated a limited number of inflammatory markers affected by cardiac surgery with a sternotomy in adults, with no consensus on the inflammatory effect of sternotomy (12, 13). Importantly, there have not been any pediatric studies investigating this topic, while adult studies have primarily focused on the inflammatory response for the entire cardiac surgery, either comparing mediators before sternotomy to the end of CPB or surgery and comparing surgeries with versus without sternotomy (5, 14). This study aims to describe inflammatory mediator profiles associated with sternotomy and to contextualize them within the inflammatory milieu at the end of CPB during children's heart surgery, to identify novel therapeutic targets.

## Methods

### Study design

This is *post-hoc* cohort analysis of a single-center single-arm prospective clinical trial (NCT05154864 on ClinicalTrials.gov) investigating the relationship between circulating inflammatory mediators and post-operative clinical outcomes in pediatric patients (<30kg) undergoing cardiac surgery with CPB and continuous ultrafiltration (11). The exposure of interest in this report is sternotomy in pediatric cardiac surgery. The outcomes of interest are the concentrations of inflammatory mediators (complement, cytokines, chemokines, and adhesion molecules) before and after sternotomy, as well as the differences between the two time points. A comparative analysis was conducted between the sternotomy and CPB phases, and a within-individual pairwise analysis examined changes in inflammatory mediators over these time periods. Written informed consent was obtained from substitute decision-makers for all participants under a protocol approved by the IWK Health Centre Research Ethics Board (<#>1024869) on November 21, 2019.

### Study participants

Patients were prospectively enrolled and completed the study protocol between August 2020 and June 2021 at the IWK Health Centre in Halifax, Nova Scotia, Canada. Exclusion criteria included: absence of written consent, known severe hematologic abnormality, genetic syndrome with severe multi-organ involvement, immunodeficiency syndrome and severe liver disease. A total of 49 consecutive patients were screened during the study period, and 40 patients (82%) gave informed consent to participate. Nine patients (18%) were not enrolled due to a genetic syndrome with severe multi-organ abnormalities (n = 3, 6%), weight over 30 kg (n = 3, 6%), logistical process restraints (n = 2, 4%) and refusal to participate (n = 1, 2%). All 40 enrolled participants completed data collection and are included in this *post-hoc* analysis with no exclusions.

### Data collection

Patient baseline demographic and clinical information were recorded from medical records. Arterial blood samples were drawn at three specific timepoints to temporally delineate the sternotomy and, subsequently, the CPB exposure. The first sample was before sternotomy (pre-sternotomy time point A), the second sample was after sternotomy but before CPB initiation (post-sternotomy sample time point B), and the final sample was immediately after the cessation of CPB (end-CPB sample time point C). Therefore, the sternotomy phase is denoted between points A and B, the CPB phase between points B and C, and the combined phase between points A and C. These samples were collected in EDTA tubes, centrifuged for 10 minutes (500 x gravity), and the resulting plasma was extracted and subjected to a second centrifugation for 20 minutes (2500 x gravity) to yield platelet-free plasma, which was aliquoted, flash-frozen in liquid nitrogen, and stored at -80˚C. All patient samples were obtained during a volatile general anesthetic, as per standard of care.

### Immunoanalysis

*Luminex* immunoanalysis of patient samples was completed with a *Bio-Rad Bio-Plex^®^ 200* System (Hercules, United States). Thirty-three pre-specified human inflammatory factors were analyzed using multiple analysis kits including: *ThermoFisher* C3a Simplex Kit (EPX010-12282-901, Waltham, United States), *Millipore Sigma* Human Complement Magnetic Beat Panel 1 (HCMP1MAG-19K-05, Burlington, United States), *Millipore Sigma* Human Complement Magnetic Beat Panel 2 (HCMP2MAG-19K-06, Burlington, United States), *BioTechne R&D Systems* Human XL Cytokine Luminex Performance Panel (FCSTM18-21, Minneapolis, United States), *BioTechne R&D Systems* Human Magnetic Luminex Assay (LXSAHM-05, Minneapolis, United States) and *BioTechne R&D Systems* Human Magnetic Luminex Assay (LXSAHM-01, Minneapolis, United States). Bio-Rad Bio-Plex^®^ Manager™ Software 6.2 (Hercules, United States) was used to acquire the data and to perform logistic-5PL regression for all analytes. All assays were conducted according to the manufacturer's instructions.

### Statistical analysis

The changes in 33 inflammatory mediator concentrations are characterized between three time points: A) pre-sternotomy, B) post-sternotomy, and C) end-CPB. The sternotomy phase is between time points A and B, the CPB phase is between B and C, and the combined phase is between A and C. Categorical variables are reported as numbers (%), and continuous variables are presented as medians and interquartile range (IQR). A Friedman test compared mediator concentrations through the three time points in a dependent fashion. The differences in paired inflammatory mediator concentrations were calculated for each of the three phases. The median difference (MD) [95% confidence interval] was calculated using the Hodges-Lehmann estimator between timepoints A-to-B (sternotomy), and B-to-C (CPB), and A-to-C (combined). The median fold change for each inflammatory mediator concentration between time points A-to-B (sternotomy), and time points B-to-C (CPB) and time points A-to-C (combined) was calculated by (*[mediator]_time point z_ – [mediator]_time point y_/[mediator]_time point y_)* and [95% CI] estimated by 1000 nonparametric bootstrap samples with adjusted percentile interval (15).

A principal component analysis (PCA) with hierarchical clustering on principal components (HCPC) with FactoMineR was conducted to explore the relationships between dynamic mediator changes during sternotomy (16, 17). PCA transforms large multi-variable datasets into reduced forms by creating principal components which contain information and remove excessive variability of uncorrelated variables (17). HCPC builds upon the refined principal components and uses Ward's method to aggregate groups of variables with minimal variance (16, 17). Thirty-four variables were analyzed, including all 33 differences in inflammatory mediators between time points A and B, as well as sternotomy time. No imputation of missing data was performed. Mahalanobis distance was calculated to describe the differences between clusters, through all dimensions of the principal component analysis, and statistically evaluated by the Hotelling T^2^ test, F Statistic, and corresponding p-value (18). All statistical analyses were performed using R (version 4.4.2, *R Foundation for Statistical Computing*, Vienna, Austria). Statistical significance was α = 0.05.

## Results

### Patient demographics and clinical data

During the study period, 40 pediatric patients were enrolled and completed the study protocol. Baseline characteristics of the study population are summarized in **Table 1**. The majority of patients were male (58%), less than one year old (55%), and had a variety of cardiac pathologies. Fourteen patients (35%) received steroid administration, and a redo sternotomy was performed in 11 patients (28%).

**Table 1:** Patient demographics (n=40).

Clinical Information	
Sex	No. (%); Median (IQR)
Male	23 (58%)
Female	17 (42%)
Age (months)	7.3 (1.7 – 39.0)
Neonate (< 30 days)	10 (25%)
Infant (30 days – 1 year)	12 (30%)
Child (> 1 year)	18 (45%)
Weight (kg)	6.7 (4.6 – 14.9)
Body Surface Area (m^2^)	0.35 (0.27 – 0.64)
Redo Sternotomy	11 (28%)
Steroid Administration (prednisone equivalents mg/kg)	14 (35%); 12 (11 – 21)
Time Between Exposure Samples (min)	
Sternotomy (time point A – B)	104 (82 – 136)
CPB (time point B – C)	214 (163 – 275)
Combined (time point A – C)	315 (267 – 430)

Congenital heart pathology	
Ventricular Septal Defect	8 (20%)
Atrial Septal Defect	4 (10%)
Total Anomalous Pulmonary Venous Return	4 (10%)
d-Transposition of the Great Arteries	4 (10%)
Sub-Aortic Stenosis	3 (9%)
Incomplete Atrioventricular Septal Defect	2 (5%)
Right Ventricular Outflow Tract Obstruction	2 (5%)
Tetralogy of Fallot	2 (5%)
Aortic Arch Hypoplasia	1 (2%)
Aorto-Pulmonary Window and Interrupted Arch	1 (2%)
Complete Atrioventricular Septal Defect	1 (2%)
Double Outlet Right Ventricle	1 (2%)
Truncus Arteriosus	1 (2%)
Single Ventricle – Central Shunt	1 (2%)
Single Ventricle – Bidirectional Glenn	2 (5%)
Single Ventricle – Fontan	3 (9%)

### Inflammatory mediator profiles

**Figure 1:**
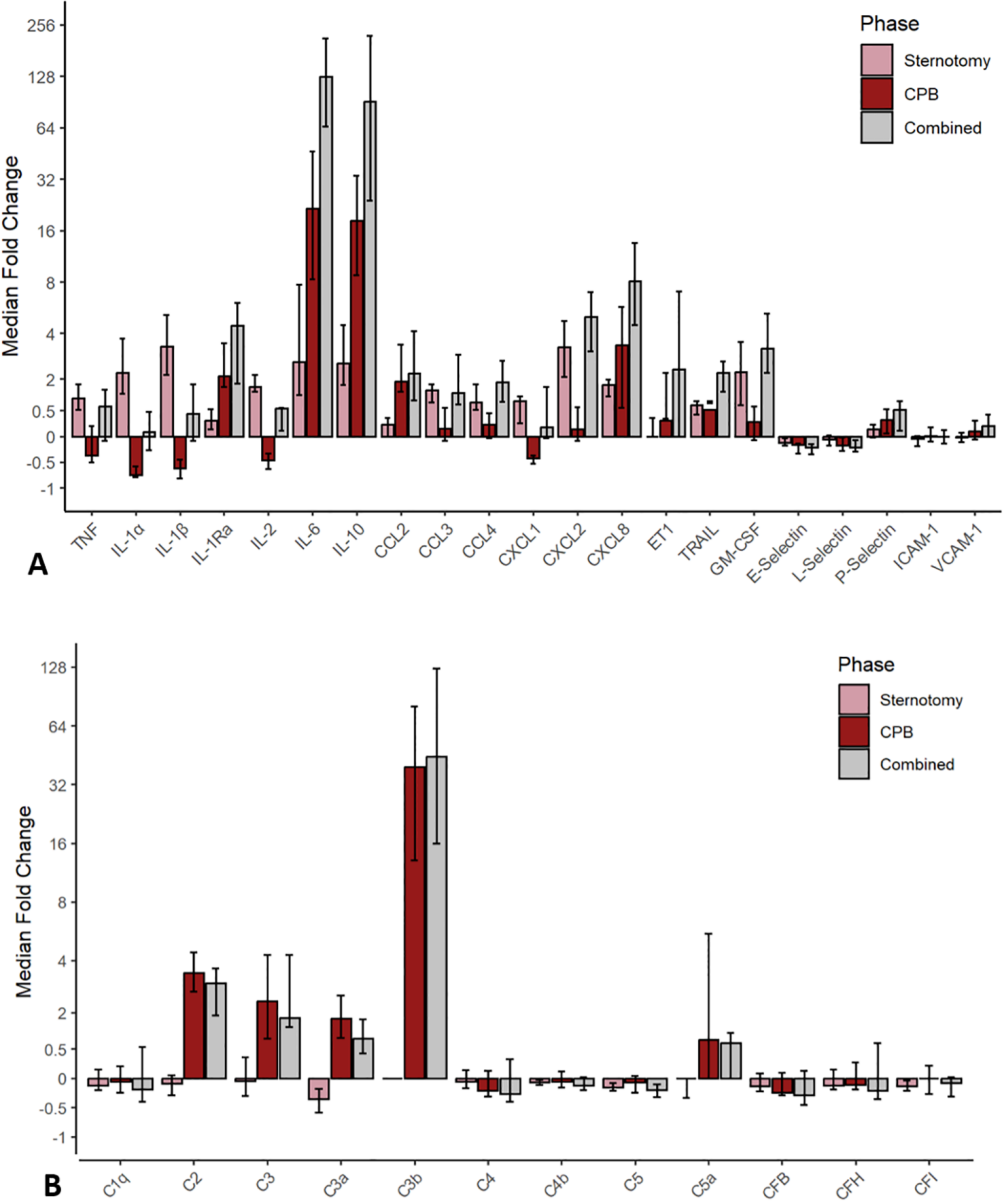
Cytokine, chemokine, and adhesion molecule **(A)** and complement **(B)** mediator median fold change [95% CI] across the sternotomy, CPB, and combined phases. CPB, cardiopulmonary bypass; C, complement; CCL, CC chemokine ligand; CF, complement factor; CXCL, CXC chemokine ligand; ET1, endothelin-1; GM-CSF, granulocyte-macrophage stimulating factor; ICAM-1, intracellular adhesion molecule 1; IL, interleukin; TNF, tumor necrosis factor; TRAIL, tumor necrosis factor-related apoptosis-inducing ligand; VCAM-1, vascular cell adhesion molecule 1.

The majority of inflammatory mediators showed dynamic changes between time points A, B and C (p < 0.05) with the exceptions of C1q (p = 0.67), C4 (p = 0.14), CFB (p = 0.12), CFH (p = 0.50), ICAM-1 (p = 0.46) and VCAM-1 (p = 0.10). The mediator concentration and pairwise comparisons with median differences (MD) [95% CI] for each mediator are listed in **Tables 2**, **3**, while the median fold changes [95% CI] of each mediator through sternotomy, CPB and combined phases are illustrated in **Figures 1A, B**.

**Table 2:** Complement mediator concentrations (n=40).

Mediator	Time point A (IQR)	Time point B (IQR)	Time point C (IQR)	Sternotomy (time point A – B)		CPB (time point B – C)		Combined (time point A – C)	
				MD [95% CI]	P-value	MD [95% CI]	P-value	MD [95% CI]	P-value
C1q (µg/ml)	55.4 (35.6 – 79.1)	47.2 (33.9 – 72.8)	53.5 (28.7 – 84.3)	-3.8 [-14.1 – 6.8]	0.45	2.6 [-10.5 – 24.1]	0.70	2.8 [-16.2 – 24.1]	0.78
C2 (µg/ml)	0.49 (0.36 – 0.69)	0.42 (0.29 – 0.57)	1.98 (1.16 – 3.00)	-0.08 [-0.16 – -0.01]	0.04	1.69 [1.33 – 2.11]	1.8 x 10^-12^	1.55 [1.18 – 1.98]	8.1 x 10^-10^
C3 (µg/ml)	14.1 (9.1 – 22.1)	15.3 (9.8 – 22.9)	55.0 (24.8 – 113.5)	-0.3 [-6.7 – 6.0]	0.91	53.1 [35.6 – 76.2]	4.5 x 10^-11^	46.3 [18.6 – 74.3]	6.2 x 10^-5^
C3a (ng/ml)	28.8 (14.9 – 41.1)	17.6 (9.0 – 26.4)	52.0 (35.0 – 60.0)	-11.4 [-19.5 – -4.7]	3.4 x 10^-4^	30.0 [24.1 – 36.5]	1.0 x 10^-7^	18.3 [11.2 – 25.5]	1.4 x 10^-4^
C3b (µg/ml)	2.8 (2.0 – 19.2)	6.6 (1.5 – 27.7)	290.0 (164.7 – 519.5)	1.0 [-17.5 – 21.4]	0.87	324.8 [230.8 – 472.7]	2.3 x10^-9^	320.2 [225.2 – 476.8]	1.3 x 10^-11^
C4 (µg/ml)	165.3 (101.4 – 233.7)	154.8 (105.5 – 218.2)	155.6 (91.1 – 214.9)	-6.6 [-30.7 – 19.5]	0.59	-8.9 [-42.7 – 33.8]	0.73	-9.4 [-59.0 – 30.7]	0.75
C4b (µg/ml)	13.7 (11.4 – 15.2)	12.2 (10.5 – 13.8)	11.6 (10.0 – 13.4)	-0.9 [-1.3 – -0.4]	1.1 x 10^-4^	-0.5 [-1.7 – 0.6]	0.40	-1.5 [-2.5 – -0.4]	0.01
C5 (µg/ml)	14.5 (10.6 – 18.7)	12.8 (10.0 – 15.0)	11.4 (8.7 – 17.1)	-2.2 [-3.1 – -1.3]	3.8 x 10^-6^	-0.6 [-2.1 – 1.1]	0.40	-3.0 [-4.4 – -1.3]	2.2 x 10^-3^
C5a (pg/ml)	33.6 (11.3 – 113.4)	20.1 (7.8 – 73.4)	109.9 (24.0 – 268.9)	-32.7 [-51.5 – 11.0]	3.1 x 10^-3^	106.4 [56.2 – 192.6]	2.2 x 10^-5^	74.8 [21.6 – 143.9]	6.3 x 10^-6^
CFB (µg/ml)	147.4 (107.6 – 214.8)	150.9 (98.8 – 185.7)	136.6 (77.3 – 182.1)	-16.0 [-41.7 – 12.6]	0.26	-9.5 [-36.2 – 27.0]	0.63	-21.6 [-61.9 – 19.5]	0.36
CFH (µg/ml)	194.4 (125.4 – 287.9)	176.3 (128.7 – 261.9)	193.6 (111.5 – 313.7)	-11.2 [-46.0 – 28.0]	0.48	10.4 [-31.9 – 74.1]	0.67	11.9 [-58.7 – 78.2]	0.73
CFI (µg/ml)	22.1 (19.0 – 38.3)	19.6 (15.2 – 28.2)	21.9 (17.9 – 27.9)	-4.0 [-7.8 – -2.2]	1.4 x 10^-4^	-1.3 [-8.7 – 4.4]	0.66	-7.1 [-15.5 – -0.5]	0.04

CI, confidence interval; CPB, cardiopulmonary bypass; C, complement; CF, complement factor; CPB, cardiopulmonary bypass; IQR, interquartile range; MD, median difference.

**Table 3:** Cytokine, chemokine and adhesion molecule concentrations (n=40).

Mediator	Time point A (IQR)	Time point B (IQR)	Time point C (IQR)	Sternotomy (time point A – B)		CPB (time point B – C)		Combined (time point A – C)	
				MD [95% CI]	P-value	MD [95% CI]	P-value	MD [95% CI]	P-value
TNF (pg/ml)	17.1 (12.5 – 20.9)	28.7 (21.6 – 37.3)	19.0 (10.7 – 76.9)	11.9 [9.4 – 14.4]	2.3 x 10^-9^	7.4 [-11.7 – 67.5]	0.74	19.6 [1.8 – 75.1]	0.01
IL-1α (pg/ml)	18.4 (7.3 – 28.7)	57.7 (42.6 – 64.9)	17.7 (7.3 – 31.4)	38.7 [32.8 – 43.9]	1.8 x 10^-12^	-38.0 [-44.8 – -31.3]	5.9 x 10^-8^	2.0 [-5.3 – 8.7]	0.68
IL-1β (pg/ml)	1.9 (0.7 – 3.0)	7.9 (5.3 – 9.7)	2.9 (1.0 – 4.6)	5.7 [4.6 – 6.9]	6.9 x 10^-8^	-5.1 [-6.3 – -3.5]	2.7 x10^-7^	1.1 [0.1 – 2.2]	0.04
IL-1Ra (ng/ml)	0.31 (0.22 – 0.48)	0.37 (0.30 – 0.56)	1.86 (0.88 – 6.25)	0.09 [0.04 – 0.16]	3.6 x 10^-3^	1.71 [1.03 – 4.92]	5.3 x 10^-7^	2.04 [1.18 – 5.39]	5.5 x 10^-8^
IL-2 (pg/ml)	4.4 (2.2 – 7.1)	12.4 (8.3 – 15.5)	5.7 (4.3 – 9.8)	7.5 [6.3 – 8.7]	5.5 x 10^-8^	-5.1 [-6.9 – -3.0]	2.5 x 10^-4^	2.5 [1.3 – 4.3]	1.7 x 10^-3^
IL-6 (pg/ml)	1.6 (0.4 – 7.4)	10.2 (4.6 – 23.4)	245.5 (99.7 – 754.2)	9.4 [5.7 – 14.5]	1.2 x 10^-3^	375.1 [179.2 – 563.8]	1.8 x 10^-12^	371.6 [189.8 – 570.3]	1.8 x 10^-12^
IL-10 (ng/ml)	0.02 (0.01 – 0.04)	0.08 (0.04 – 0.12)	1.4 (0.26 – 4.12)	0.06 [0.04 – 0.08]	6.9 x 10^-4^	2.13 [1.24 – 3.92]	3.6 x 10^-12^	2.19 [1.27 – 3.99]	3.6 x 10^-12^
CCL2 (ng/ml)	177.8 (130.7 – 241.9)	202.0 (166.8 – 261.9)	601.9 (340.7 – 1352.4)	36.8 [9.2 – 67.9]	8.6 x 10^-3^	611.0 [325.9 – 1003]	8.1 x 10^-10^	677.8 [337.2 – 1092]	6.0 x 10^-11^
CCL3 (pg/ml)	17.7 (14.1 – 24.8)	31.5 (26.5 – 37.4)	33.5 (27.2 – 104.7)	14.1 [11.5 – 16.6]	1.2 x 10^-8^	19.1 [2.1 – 118.3]	0.02	37.5 [20.1 – 150.2]	5.9 x 10^-7^
CCL4 (ng/ml)	0.24 (0.17 – 0.33)	0.52 (0.47 – 0.64)	0.60 (0.47 – 0.95)	0.31 [0.27 – 0.35]	5.9 x 10^-8^	0.16 [0.03 – 0.43]	0.01	0.46 [0.33 – 0.71]	3.6 x 10^-12^
CXCL1 (pg/ml)	79.5 (60.0 – 119.6)	171.4 (134.8 – 219.7)	96.8 (66.9 – 165.7)	96.5 [78.6 – 121.8]	3.6 x 10^-12^	-16.0 [-91.0 – -37.0]	4.3 x 10^-4^	36.0 [9.5 – 67.6]	8.7 x 10^-3^
CXCL2 (ng/ml)	0.42 (0.27 – 0.63)	1.96 (1.13 – 2.72)	2.33 (1.50 – 3.35)	1.53 [1.22 – 1.95]	1.8 x 10^-12^	0.43 [-0.23 – 1.20]	0.16	2.02 [1.45 – 2.89]	1.8 x 10^-11^
CXCL8 (pg/ml)	5.5 (3.9 – 9.5)	11.1 (8.6 – 17.6)	57.8 (30.0 – 149.3)	6.2 [4.7 – 9.9]	3.1 x 10^-10^	63.7 [31.3 – 108.7]	4.3 x 10^-9^	72.4 [43.0 – 116.4]	1.8 x 10^-12^
ET-1 (pg/ml)	0.56 (0.36 – 1.21)	1.17 (0.36 – 3.29)	1.75 (0.89 – 5.83)	2.8 [1.5 – 4.3]	2.1 x 10^-3^	2.72 [0.86 – 5.56]	0.01	4.6 [2.7 – 8.2]	9.1 x 10^-5^
GM-CSF (pg/ml)	4.2 (1.8 – 10.6)	19.4 (11.7 – 26.6)	24.0 (17.4 – 28.2)	14.1 [10.6 – 17.6]	1.7 x 10^-5^	5.8 [0.6 – 11.4]	0.03	18.1 [13.9 – 23.1]	1.0 x 10^-7^
TRAIL (pg/ml)	93.8 (79.1 – 145.9)	165.1 (124.0 – 211.7)	319.5 (224.7 – 460.2)	55.8 [39.9 – 70.7]	8.2 x 10^-3^	176.9 [127.6 – 229.1]	3.9 x 10^-7^	222.5 [174.9 – 276.9]	3.6 x 10^-12^
E-Selectin (ng/ml)	58.3 (34.8 – 76.9)	52.0 (32.7 – 65.5)	38.7 (27.7 – 49.3)	-4.5 [-8.8 – 0.2]	0.06	-12.6 [-18.5 – -6.4]	6.7 x 10^-5^	-14.9 [-22.7 – -8.2]	1.9 x 10^-5^
L-Selectin (ng/ml)	625.7 (391.2 – 886.9)	582.0 (404.9 – 868.8)	559.9 (346.6 – 703.0)	-47.6 [-98.4 – 3.1]	0.06	-92.3 [-161.5 – -28.5]	2.9 x 10^-3^	-122.9 [-201.0 – -53.3]	5.1 x 10^-4^
P-Selectin (ng/ml)	31.2 (24.1 – 39.6)	35.8 (27.3 – 46.2)	47.3 (35.8 – 54.4)	3.6 [0.6 – 6.8]	0.02	9.6 [5.0 – 14.9]	1.9 x 10^-4^	13.4 [8.6 – 19.1]	1.4 x 10^-5^
ICAM-1 (ng/ml)	298.1 (194.0 – 369.5)	276.9 (190.5 – 387.6)	272.9 (191.3 – 408.6)	-15.8 [-36.8 – 8.6]	0.17	7.8 [-22.8 – 37.7]	0.62	-1.0 [-34.8 – 33.5]	0.97
VCAM-1 (ng/ml)	72.4 (60.7 – 143.6)	92.4 (63.7 – 132.9)	100.1 (78.3 – 152.3)	0.0 [-7.7 – 10.1]	0.99	10.0 [-4.8 – 22.3]	0.14	12.6 [-6.0 – 26.3]	0.17

CI, confidence interval; CPB, cardiopulmonary bypass; CCL, CC chemokine ligand; CXCL, CXC chemokine ligand; ET-1, endothelin-1; GM-CSF, granulocyte-macrophage colony-stimulating factor; ICAM-1, intracellular adhesion molecule 1; IL, interleukin; IQR, interquartile range; MD, median difference; TNF, tumor necrosis factor; TRAIL, tumor necrosis factor-related apoptosis-inducing ligand; VCAM-1, vascular cell adhesion molecule 1.

### Sternotomy phase

The sternotomy duration between points A and B was 104 (82–136) minutes. The cytokines TNF, IL-1α, IL-1β, IL-2, IL-6, IL-10, IL-1Ra, TRAIL, GM-CSF and the chemokines CCL2, CCL3, CCL4, CXCL1, CXCL2, CXCL8, as well as ET1 and P-selectin increased (p < 0.05) between the time points A and B. The complement factors C2, C3a, C4b, C5, C5a and CFI showed significant reductions (p < 0.05) while C1q, C3, C3b, C4, CFB, CFH, E-selectin, L-selectin, ICAM-1 and VCAM-1 did not change (p > 0.05). IL-1β (3.3x median fold increase), CXCL2 (3.3x), IL-6 (2.6x), IL-10 (2.6x), GM-CSF (2.3x), IL-1α (2.2x) and IL-2 (1.7x) showed the largest dynamic increases. Complement mediators were static or decreased, with magnitudes ranging from C5a (0.0x median fold change) to C3a (-0.4x median fold decrease). There was no difference in mediator changes between male and female sex during sternotomy (p > 0.05). A linear correlation was found between sternotomy time and mediator change over the period in IL-6 (Rho = 0.49; p = 0.001), IL-1Ra (Rho = 0.44; p = 0.005), TNF (Rho = 0.038; p = 0.02) and TRAIL (Rho = 0.32; p = 0.04). All other mediator changes did not correlate with sternotomy time.

**Figure 2:**
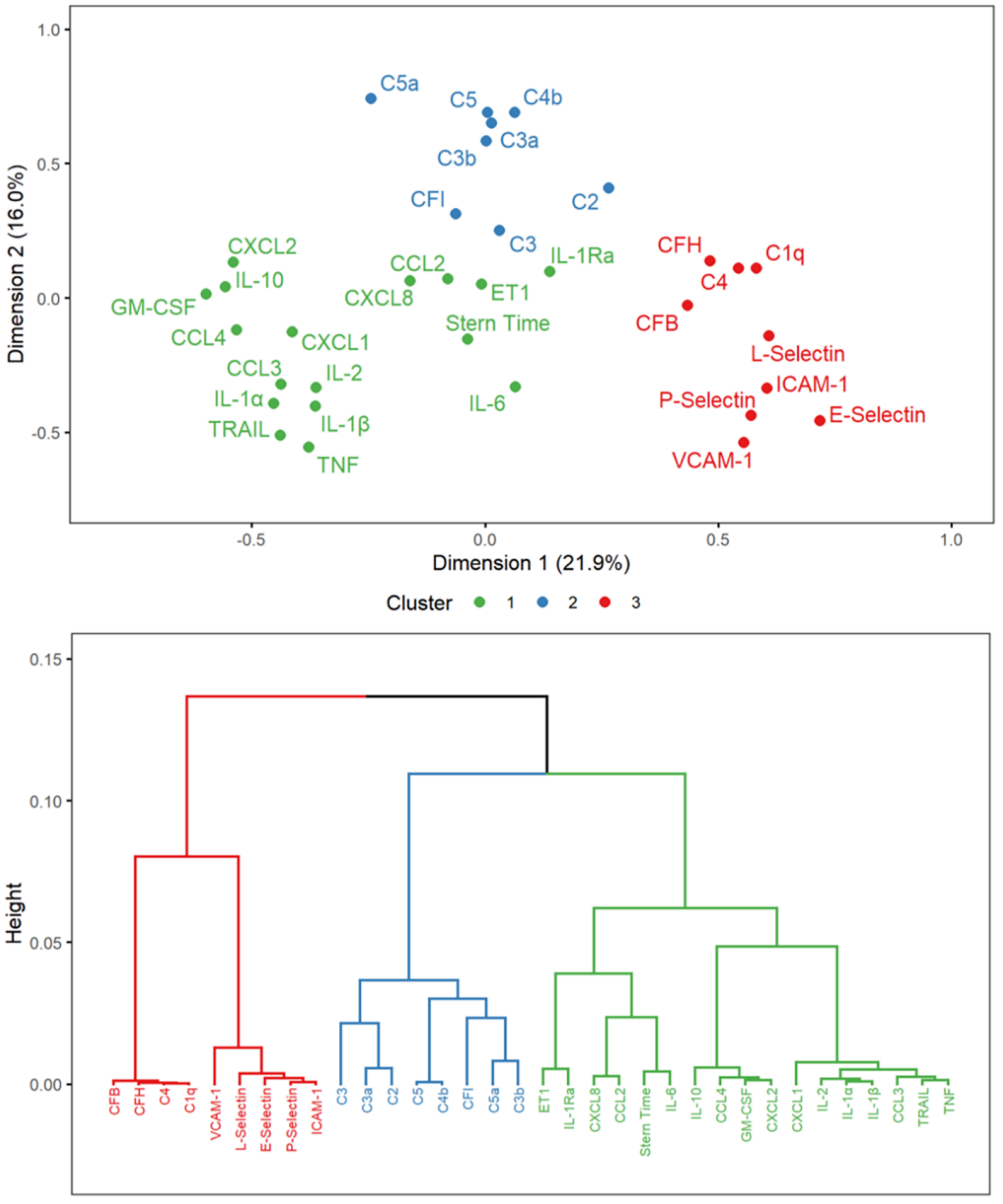
Hierarchical clustering on principal components factor map and dendrogram for mediator changes over sternotomy. Mediator variables represent changes between time points A and B during the sternotomy. Cluster 1 & 2 (p = 5.0 x 10^-4^), cluster 1 & 3 (p = 3.3 x 10^-4^), and cluster 2 & 3 (p = 0.03) are statistically different than one another. C, complement; CCL, CC chemokine ligand; CF, complement factor; CXCL, CXC chemokine ligand; ET1, endothelin-1; GM-CSF, granulocyte-macrophage stimulating factor; ICAM-1, intracellular adhesion molecule 1; IL, interleukin; Stern Time, sternotomy time; TNF, tumor necrosis factor; TRAIL, tumor necrosis factor-related apoptosis-inducing ligand; VCAM-1, vascular cell adhesion molecule 1.

The PCA revealed 10 dimensions that accounted for 84% of the variance in the dataset; dimensions 1 and 2 accounted for 21.9% and 16.0% of the variance, respectively, while the HCPC revealed 3 clusters, as illustrated in **Figure 2**. Cluster 1 consisted of sternotomy time along with all cytokine and chemokine mediators. Cluster 2 included the complement mediators C2, C3, C3a, C3b, C4b, C5, C5a and CFI. Cluster 3 was a mix of complement mediators C1q, C4, CFB and CFH, along with all adhesion molecules. Cluster 1 was statistically different than cluster 2 (Mahalanobis distance = 21.5; T^2^ = 116.8; F = 7.1; p = 5.0 x 10^-4^), and cluster 3 (Mahalanobis distance = 19.8; T^2^ = 116.3; F = 7.3; p = 3.3 x 10^-4^), while cluster 2 was statistically different than cluster 3 (Mahalanobis distance = 27.4; T^2^ = 116.2; F = 4.6; p = 0.03). The PCA and HCPC did not cluster individuals by age group based on changes in inflammatory mediators during sternotomy.

### Cardiopulmonary bypass phase

The cytokines IL-2, IL-6, IL-10, IL-1Ra, TRAIL, GM-CSF, the chemokines CCL2, CCL3, CCL4, CXCL1, CXCL8, ET1 and P-selectin continued to rise and again increased significantly (p < 0.05) between the time points B and C. Additionally, complement factors C2, C3, C3a, C3b, C5a also increased significantly (p < 0.05). IL-1α, IL1β, IL-2, CXCL1, E-selectin and L-selectin decreased significantly (p < 0.05) while the other mediators did not change (p > 0.05). C3b (39.3x median fold increase), IL-6 (21.5x), IL-10 (18.3x) were the most responsive, followed by C2 (3.4x), CXCL8 (3.4x), C3 (2.4x), IL-1Ra (2.1x) and the complement anaphylatoxins C3a (1.8x) and C5a (1.2x). IL-1α (-0.7x median fold decrease), IL-1β (-0.6x), IL-2 (-0.5x), CXCL1 (-0.4x) and TNF (-0.4x) showed the largest dynamic decreases through the phase.

### Combined phases

**Figure 3:**
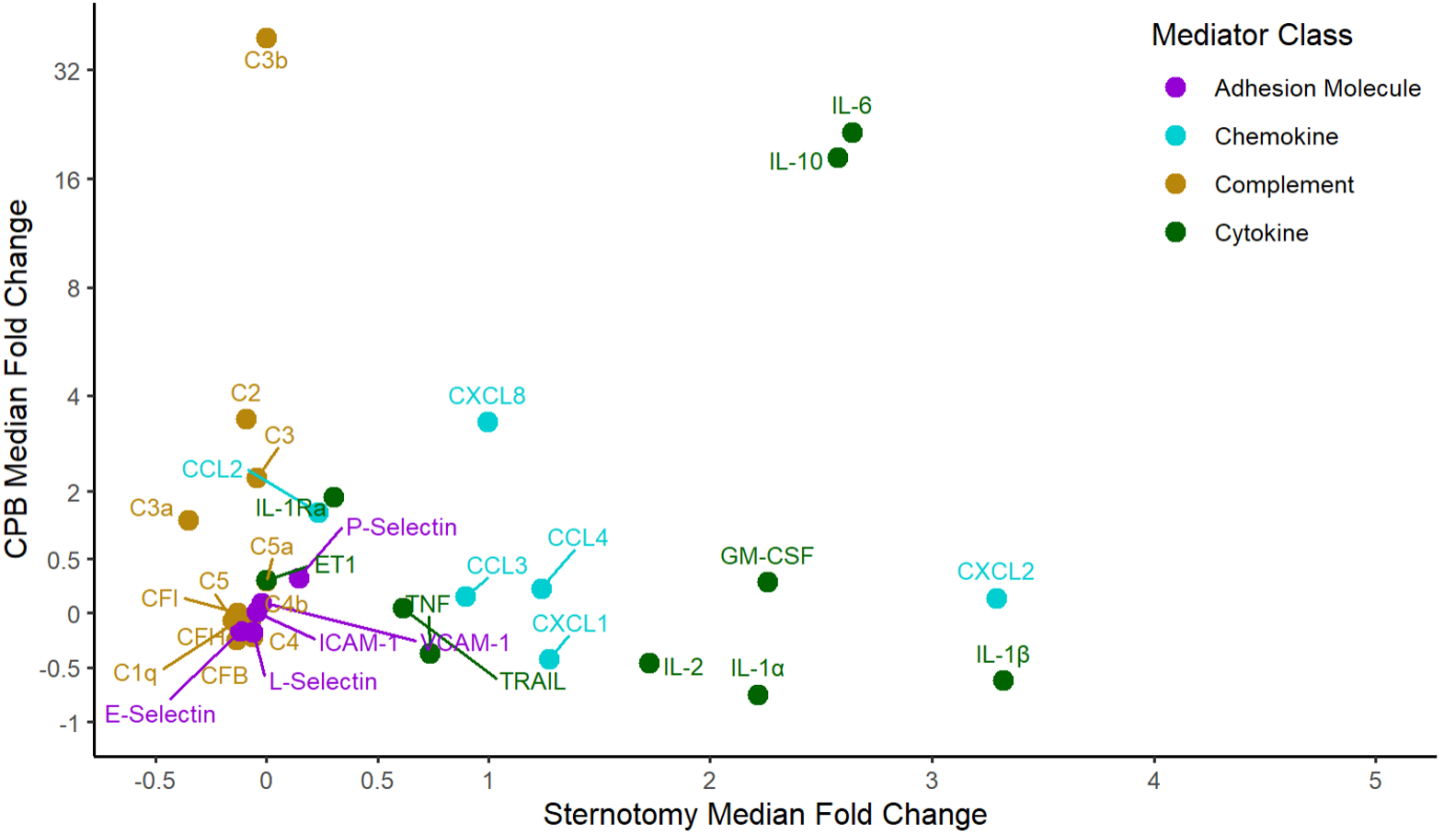
Median fold changes between sternotomy and CPB. CPB, cardiopulmonary bypass; C, complement; CCL, CC chemokine ligand; CF, complement factor; CXCL, CXC chemokine ligand; ET1, endothelin-1; GM-CSF, granulocyte-macrophage stimulating factor; ICAM-1, intracellular adhesion molecule 1; IL, interleukin; TNF, tumor necrosis factor; TRAIL, tumor necrosis factor-related apoptosis-inducing ligand; VCAM-1, vascular cell adhesion molecule 1.

The cytokines TNF, IL-1β, IL-2, IL-6, IL-10, IL-1Ra TRAIL, GM-CSF, the chemokines CCL2, CCL3, CCL4, CXCL1, CXCL2, CXCL8, the complement factors C2, C3, C3a, C3b, C5a as well as ET1 and P-selectin increased significantly (p < 0.05) between the time points A and C. C4b, C5, CFI, L-selectin and E-selectin decreased significantly (p < 0.05) while IL-1α, ICAM-1 and VCAM-1 did not change (p > 0.05). IL-6 (126.9x median fold increase), IL-10 (91.2x), C3b (44.6x), CXCL8 (8.1x), CXCL2 (5.0x) and IL-1Ra (4.4x) showed the largest magnitude of concentration changes while C3a (0.7x), C5a (0.6x), TNF (0.6x), IL-1β (0.4x) and IL-1α (0.1x) also increased. Compared to baseline, CFB (-0.3x median fold decrease), C4 (-0.3x), E-selectin (-0.2x) and L-selectin (-0.2x) had the largest magnitude of reduction from baseline. The relative dynamics of each mediator are plotted in **Figure 3** to illustrate the contribution of sternotomy and CPB to the combined median fold change. The cytokines and chemokines TNF, IL-1α, IL-1β, TRAIL, CCL3, CCL4, CXCL1, CXCL2, and GM-CSF were predominantly active during sternotomy, rather than during CPB.

## Discussion

Sternotomy has an active and distinct inflammatory profile during pediatric cardiac surgery. Our findings indicate that incisional trauma is associated with significant increases in circulating cytokine and chemokine concentrations, but not in any complement factors, because the patient's circulation has not yet been exposed to the non-endothelialized artificial surface of CPB. The sternotomy findings are contrasted against the significant CPB complement-mediated role in the inflammatory response, consistent with previous reports (11, 19). Several cytokines and chemokines – TNF, IL-1α, IL-1β, IL-2, TRAIL, CCL3, CCL4, CXCL1, CXCL2 and GM-CSF – were unique to sternotomy. IL-6, CXCL8, IL-10 and IL-1Ra were relevant to both sternotomy and CPB, exhibiting a multiplicative effect, as these mediators had a substantially higher fold increase during CPB compared to sternotomy. The continuous cytokine and chemokine production could relate to ongoing processes from sternotomy tissue injury, downstream effects from the complement reaction and also myocardial ischemia reperfusion during CPB (1, 2). C3a, C3b, IL-6 and CXCL8 are known to produce and stimulate direct and systemic neutrophil activation and release of pro-inflammatory cytokines, which directly facilitate endothelial leak syndrome, neutrophil recruitment, tissue edema, and subsequent injury that contributes to end-organ dysfunction in the post-operative period (1, 10, 11).

P-selectin was one of the adhesion molecules that increased significantly with sternotomy. This mediator plays a key role in wound hemostasis, in addition to leukocyte recruitment, explaining the immediate relevance to sternotomy (20). Other adhesion molecules are elicited in a transcription-dependent way by cytokines and chemokines and thus require several hours to achieve peak expression (21, 22). Therefore, this might explain why the other leukocyte adhesion molecules did not change significantly, as these are downstream mediators in the inflammatory cascade that would likely be elevated later in the post-operative period.

This was the first investigation to comprehensively characterize the dynamics of inflammatory mediators of sternotomy in children. Prior studies in adults primarily focused on the inflammatory response throughout the cardiac procedure, comparing mediators measured before sternotomy with those at the end of CPB (5, 14). A few adult studies have data on a limited number of inflammatory mediators affected by sternotomy, most of which contradict our results. Three studies reported no change in IL-6 concentrations during sternotomy (12, 23, 24), whereas two others reported a decrease (25, 26), in contrast to the robust 2.6x median fold increase observed here. Our results demonstrate that IL-6 concentration increases over time, a finding that may differ across studies. Additionally, the latter studies observed no changes in TNF and CXCL8, whereas our study demonstrated statistically significant increases in these mediators (27). C3a and C5b-9 were found to be active after anterolateral thoracotomy for CABG, which again conflicts with our observations (13). ET1 was the only mediator that showed a similar profile between pediatric and adults, as a single study reported dynamic increases over sternotomy in a CABG population (27). Allowing for differences in patient populations and study designs across these prior reports, one might conclude that the inflammatory response to sternotomy may differ between children and adults. However, this contrast should be interpreted with caution for several reasons. First, the adult studies were relatively small, with the largest being 30 patients divided into subgroups, and were not specifically designed to understand the inflammatory profile of sternotomy, as this report does. Secondly, the concept of immunosenescence is relevant to the elderly and comorbid patient populations (28). Third, pediatric congenital heart disease and adult acquired cardiac disease are distinct patient populations in terms of age and pathophysiology. Finally, the modern *Luminex* technology used in this study is more sensitive than historical enzyme-linked immunosorbent assay (ELISA) approaches.

IL-1β was the most active mediator during sternotomy, although the related mediators TNF and IL-1α also showed significant increases in circulation. IL-1α is released during cellular injury; TNF expression is primarily induced within activated macrophages, while IL-1β requires transcription and cleavage by caspase for biological activity (22, 29). These findings support current immunological understanding that, together, these are early pro-inflammatory signals produced mainly by macrophages, monocytes, and neutrophils in response to tissue trauma and damage-associated molecular patterns (30). Specifically, they induce the production of other innate cytokines and chemokines that activate macrophage and neutrophil machinery required for healing (29, 30). Interestingly, IL-1α, IL-1β, and TNF were predominantly active during sternotomy and inactive during CPB with continuous ultrafiltration, during which their concentrations decreased.

CXCL2 showed the second-largest median fold increase during sternotomy in our study. CXCL1 and CXCL8 also showed significant increases in concentration over the course of the sternotomy. These chemokines are all involved in the primary response to the inflammatory phase of wound healing and angiogenesis (31). In early phases of wound healing after tissue injury, this group of chemokines is released to recruit inflammatory cells, including neutrophils predominantly and macrophages, and later on, it mediates the proliferation phase of the wound healing response (31). Consistent with previous adult sternotomy reporting, we also observed GM-CSF concentration increase with sternotomy, as it is known to be upregulated in injured skin and helps orchestrate the proliferation of endothelial cells for restorative angiogenesis (7, 32).

The profiles of IL-6, CXCL8, IL-1Ra and IL-10 were unique from other mediators assessed in this study, as they consistently increased through the sternotomy and CPB phase, perhaps a sequential stimulation by two different stimuli (1, 10). IL-6 is a pro-inflammatory mediator released in response to tissue injury or other immunologic stimuli. Elevated concentrations after surgery and trauma are necessary for the timely resolution of wound healing by regulating leukocyte infiltration, angiogenesis, and collagen deposition (33). Consistent with our results of IL-6 production through sternotomy, other studies have detected IL-6 within 90 minutes of skin incision, lasting for several hours, and concentrations were related to the extent of the surgically induced trauma during elective procedures (34, 35). The anti-inflammatory mediator IL-1Ra is tightly coupled to pro-inflammatory cytokine production, primarily the IL-1 family of mediators, to prevent a hyper-inflammatory state and ultimately return the system to homeostasis (36). IL-10 is another anti-inflammatory mediator with a major role in the tissue repair process (37), observed to be stimulated by the sternotomy. IL-10 also has a vital role in inflammatory resolution in a variety of models of tissue repair, including cutaneous wound healing and in re-establishing tissue integrity after injury in multiple models of skin and myocardial injury (37).

**Figure 4:**
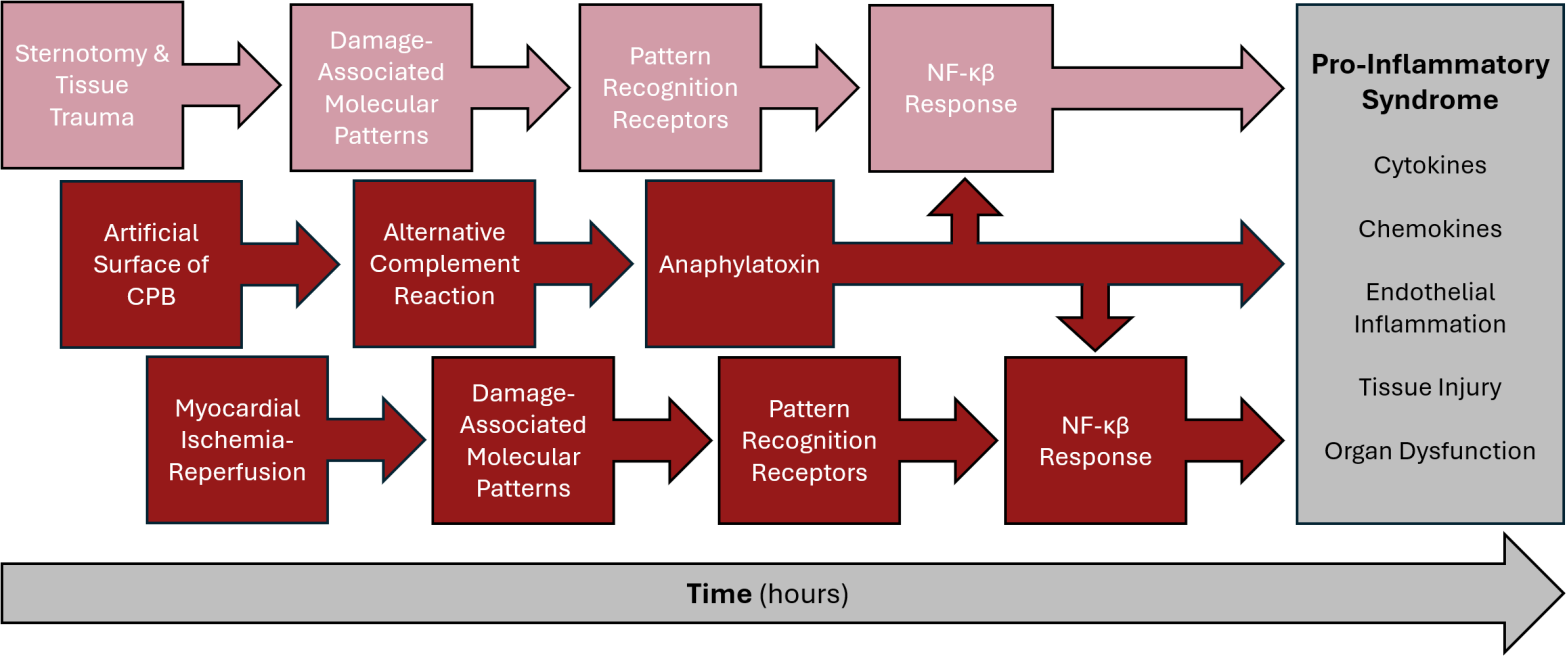
Proposed inflammatory cascade through cardiac surgery with CPB. NF-κB, nuclear factor kappa beta.

The collection of active pro-inflammatory cytokines and chemokines observed during the sternotomy phase is characterized by their downstream NF-κβ-mediated responses (7, 8, 38). Innate immune cells resident in the skin and connective tissue include macrophages, dendritic cells and mast cells, which monitor damage-associated molecular patterns (DAMPs) through pattern recognition receptors (PRRs) (38–40). Upon stimulation, these surveilling leukocytes initiate the NF-κβ response observed in this sternotomy response with cascading TNF, IL-1, IL-6, IL-1Ra and IL-10, along with a host of chemokines. Interestingly, there was virtually no complement activation during the sternotomy period, which was limited to CPB exposure. Altogether, the multiple immune stimuli of tissue trauma, CPB non-endothelialized circuit, and myocardial ischemia-reperfusion, among others, culminate in a pro-inflammatory systemic response (**Figure 4**) (1, 11).

IL-2 is traditionally described as an adaptive mediator with pleiotropic effects on T-cell function, which is largely unrelated to non-specific adaptive responses during cardiac surgery (41, 42). IL-2 has been sparsely studied during the proliferative and remodeling phases of wound healing, but has been measured in the local wound environment and in the systemic circulation in the days following tissue injury to orchestrate keratinocytes, fibroblasts and endothelial cells (43, 44). For the first time, our results indicate that circulating IL-2 concentrations increase substantially (1.7x median fold increase) immediately after the sternotomy tissue trauma. IL-2 is known to be produced by activated dendritic, mast cells and macrophages, which are stimulated during incisional damage and trauma and could explain these unique observations (42). IL-2 may have an unexplored innate immune function in the acute setting of tissue injury, particularly given that the majority of data in this study arises from immunologically immature neonates and infants. This postulated mechanism requires dedicated investigation and validation.

The authors recognize that this descriptive study is not without limitations. First, the study population was heterogeneous across several variables relevant to systemic inflammation, including patient age, congenital heart disease pathology, steroid administration, type of operative repair, CPB time, myocardial ischemia-reperfusion time, intraoperative transfusion, and hypothermia. The sample size of 40 is prohibitive for subgroup analyses by age group or for interrogating these specific perioperative variables relative to immunologic response. The impact of heterogeneity in patient baseline demographics was partially mitigated by including paired within-individual comparisons through the sternotomy and CPB phases. Second, the data are drawn from a single center, which limits generalizability. In particular, the results should be interpreted in the context of pediatric CPB with continuous ultrafiltration, where cytokines, chemokines, and complement anaphylatoxins C3a and C5a are known to be removed from the circulation (45, 46). Mediator changes throughout CPB exposure could theoretically exceed the values reported here if ultrafiltration were not used.

In conclusion, sternotomy during pediatric cardiac surgery is a major immunologic stimulus and primarily results in increased expression of circulating pro- and anti-inflammatory cytokines and chemokines – TNF, IL-1α, IL-1β, IL-2, TRAIL, CCL3, CCL4, CXCL1, CXCL2, and GM-CSF – through an NF-κB response to tissue trauma. In contrast, CPB primarily activates the complement system and further increases specific cytokines and chemokines, including IL-6, CXCL8, IL-1Ra, and IL-10. CPB and, perhaps, the myocardial ischemia-reperfusion reaction during CPB appear to make a dominant contribution to these mediators versus sternotomy. These results clearly delineate two temporally distinct, and perhaps synergistic, immunologic stimuli during pediatric cardiac surgery and lay the foundation for exploring innovative therapeutic options to dampen inflammation and enhance post-operative recovery in children with congenital heart disease.

## Data Availability

The datasets generated and/or analyzed during the study are not publicly available due to patient confidentiality but are available from the corresponding author upon reasonable request.
